# Virus neutralization assays for human respiratory syncytial virus using airway organoids

**DOI:** 10.1007/s00018-024-05307-y

**Published:** 2024-06-17

**Authors:** Laura L.A. van Dijk, Laurine C. Rijsbergen, Bruno Tello Rubio, Katharina S. Schmitz, Lennert Gommers, Anouskha D. Comvalius, Alexander Havelaar, Geert van Amerongen, Rutger Schepp, Mart M. Lamers, Corine H. GeurtsvanKessel, Bart L. Haagmans, Rob van Binnendijk, Rik L. de Swart, Rory D. de Vries

**Affiliations:** 1 Department of Viroscience, Erasmus MC, University Medical Centre Rotterdam, Rotterdam, the Netherlands; 2 Center of Infectious Disease Control, National Institute of Public Health and the Environment (RIVM), Bilthoven, the Netherlands; 3 Present Address: Programme in Emerging Infectious Diseases, Duke-NUS Medical School, Singapore, Singapore

**Keywords:** Neutralization, Primary_cells, Organoids, Respiratory_syncytial_virus, Immunity

## Abstract

Neutralizing antibodies are considered a correlate of protection against severe human respiratory syncytial virus (HRSV) disease. Currently, HRSV neutralization assays are performed on immortalized cell lines like Vero or A549 cells. It is known that assays on these cell lines exclusively detect neutralizing antibodies (nAbs) directed to the fusion (F) protein. For the detection of nAbs directed to the glycoprotein (G), ciliated epithelial cells expressing the cellular receptor CX3CR1 are required, but generation of primary cell cultures is expensive and labor-intensive. Here, we developed a high-throughput neutralization assay based on the interaction between clinically relevant HRSV grown on primary cells with ciliated epithelial cells, and validated this assay using a panel of infant sera. To develop the high-throughput neutralization assay, we established a culture of differentiated apical-out airway organoids (Ap-O AO). CX3CR1 expression was confirmed, and both F- and G-specific monoclonal antibodies neutralized HRSV in the Ap-O AO. In a side-by-side neutralization assay on Vero cells and Ap-O AO, neutralizing antibody levels in sera from 125 infants correlated well, although titers on Ap-O AO were consistently lower. We speculate that these lower titers might be an actual reflection of the neutralizing antibody capacity in vivo. The organoid-based neutralization assay described here holds promise for further characterization of correlates of protection against HRSV disease.

**Supplementary Information:**

The online version contains supplementary material available at 10.1007/s00018-024-05307-y.

## Introduction

Human respiratory syncytial virus (HRSV) is a leading cause of severe lower respiratory tract disease in infants, immunocompromised adults and frail elderly [1, 2]. In the upper respiratory tract, HRSV primarily infects ciliated epithelial cells using two viral glycoproteins for attachment and entry: the attachment (G) protein and the fusion (F) [3–5]. Although multiple cellular HRSV receptors have been described, it is thought that engagement of CX3 chemokine receptor 1 (CX3CR1) by the HRSV G protein, followed by conformational changes in the F protein, leads to fusion at the cell membrane and subsequent entry into ciliated epithelial cells [5]. Two monoclonal antibodies (moAb) targeting the F protein are available for prophylactic treatment of infants, Palivizumab and Nirsevimab [6–9]. Recently, two pre-fusion F protein-based vaccines were licensed for use in the elderly, Arexvy (GSK) and Abrysvo (Pfizer). Abrysvo was also approved for women during pregnancy [10]. Interestingly, none of the prophylactic interventions involve induction of an immune response targeting the G protein.

A recent model-based meta-analysis showed that high serum levels of HRSV-specific neutralizing antibodies are a correlate of protection against HRSV disease [11, 12]. Measuring HRSV-specific neutralizing antibodies is therefore key in assessing HRSV immune response and vaccine immunogenicity. Virus neutralization assays (VNA) employed to measure this include classical plaque reduction neutralization tests (PRNT), endpoint VNA, or focus reduction neutralization tests (FRNT), using either staining of foci of infection, flow cytometry or qPCR as readouts [13–20]. However, all these assays rely on the use of continuous cell lines, which do not represent the natural target cells of HRSV, nor do they express the entry receptor CX3CR1 [4, 21–24]. Several other HRSV receptors in cell lines have been described, which can be used by either the F or G protein [4]. As a result, HRSV can enter cell lines that do not express CX3CR1, and can even enter without a functional G protein [25]. Therefore, VNA on cell lines exclusively detect antibodies targeting the F protein. VNA in differentiated human airway epithelial cells grown at air-liquid interface (ALI) have been previously described [23, 26–29] and were shown to detect both HRSV F- and G-specific antibodies, but these assays are expensive, labor-intensive, and do not allow for the high-throughput assessments often required in clinical trials.

It has been reported that HRSV virions produced in cell lines have a lower molecular weight G protein than virions produced on differentiated human airway epithelial (HAE) cultures, probably due to differences in glycosylation patterns [30]. This alternative glycosylation of the G protein could affect the binding of G-specific antibodies to virus-particles. Therefore, the use of HRSV stocks grown on cell lines could lead to different neutralization patterns when compared to the use of HRSV stocks grown on primary respiratory epithelial cells. For accurate results in a VNA, it could be crucial to grow HRSV on its natural target cells and perform the VNA on homologous cells.

Airway organoid (AO)-based in vitro models are rapidly evolving. Culture of airway organoids in an extracellular matrix results in differentiated 3D structures with the apical surface on the inside, which are therefore poorly susceptible to respiratory virus infections [31]. Generating these 3D structures ‘inside-out’ results in apical-out airway organoids (Ap-O AO) [32], leading to increased susceptibility to respiratory viruses that enter ciliated epithelial cells at the apical side, like HRSV. These cells achieve more rapid differentiation into ciliated airway epithelial cells and therefore show great promise for use in high-throughput assays. The potential for high-throughput production of ciliated epithelial cells in combination with susceptibility to HRSV, make this model an appropriate alternative for detection of F- and G-specific neutralizing antibodies.

Here, we developed a VNA that can measure both F- and G-specific neutralizing antibodies using Ap-O AO in combination with clinical-based HRSV-A and HRSV-B expressing a fluorescent reporter protein grown in primary airway cells. We validated our novel assay with sera from HRSV-A or -B infected ferrets, and sera from infants who most likely experienced a single HRSV infection. This VNA further supports moAb development against the HRSV G protein, aids in the assessment of vaccine immunogenicity, and could lead to fine-tuning of correlates of protection against HRSV disease.

## Materials and methods

### Cells

Vero and HEp-2 cells were grown in Dulbecco’s modified Eagle’s medium (DMEM; Invitrogen; Thermo Fisher Scientific) supplemented with 10% fetal bovine serum (v/v) and 100 IU penicillin + 100 µg streptomycin (Westburg) and 2 mM glutamine (BioProduct) in 5% CO2 at 37 °C. AO at ALI were grown as described previously [31, 33]. In some experiments, a notch-inhibitor (DAPT, Tocris, cat 2634) was added at 5 µM, 10–14 days after transfer to ALI, for 7–10 days to direct differentiation towards ciliated epithelial cells. AO at ALI cultures were differentiated for 4–6 weeks, whereas AO at ALI^DAPT^ cultures were differentiated for 2–4 weeks. Ap-O AO were generated according to manufacturer’s protocol, except for the origin of the cells [34]. In short, bronchial AO were taken out of Matrigel and mechanically disrupted to single cells in a TrypleE suspension. Approximately 120.000 cells in 1 ml Ap-O AO medium (Stemcell technologies) were added to Aggrewell plates pre-coated with anti-adherence rinsing solution (Stemcell technologies). Over the next 2–4 days, the cells aggregated into small clumps (Supplemental Fig. 1A-B). Subsequently, 3D structures were transferred to pre-coated flat bottom 24 well plates and further differentiated for 7–14 days with partial medium change every 2–3 days (Supplemental Fig. 1C). Fully differentiated Ap-O AO were seeded into 96-well U bottom plates with ~ 100 Ap-O AO per well in 50 µl Ap-O AO medium (without heparin) for subsequent assays.

### Viruses

rHRSV^A11^EGFP(5) [35] and rHRSV^B05^EGFP(5) [36] (further referred to as rHRSV-A11 and rHRSV-B05) were kindly gifted by Dr. W.P. Duprex. Stocks of these viruses and HRSV strains A2 and B05 (not EGFP strains) were grown in HEp-2 cells as previously described [37]. Virus stocks in AO cultured at ALI were grown by inoculating inserts (12-well plate) with ~ 1,000 TCID_50_, harvesting apical supernatant in DPBS supplemented with calcium and magnesium (0.9 nM MgCl_2_ and 0.49 mM CaCl_2_) at 2–4 days post-inoculation, and storage in 25% sucrose at -80ºC. Viral stocks were titrated on either HEp-2 cells, Vero cells or Ap-O AO and the results were expressed in tissue culture infectious dose- 50 (TCID_50_)/ml as calculated using the Reed & Muench method [38]. Stock titrations for each experiment were conducted using the cells as employed in that assay. Titrations on Ap-O AO were performed in 3-fold dilutions of which 50 µl virus dilution was added to 50 µl of Ap-O AO suspension in 96-well U-bottom plates. Unless stated otherwise, HEp2-grown HRSV stocks were used on the Vero FRNT, AO at ALI-grown stocks were used on the Ap-O AO VNA.

### Sera

Ferrets were inoculated intranasally with 10^5^ TCID_50_ HRSV^A2^ or HRSV^B05^. Nose and throat swabs were obtained 3 days post-inoculation and viral loads were determined by endpoint titration [38]. At 21 DPI, ferrets were euthanized and sera were collected. Animal experiments were conducted at the Erasmus MC and the study protocol was approved by the Centrale Commissie Dierproeven, permit number AVD101002017903. Sera from 125 infants below the age of 3 years (to pre-select for single infections) were selected from our diagnostic serum archive; all samples were collected for diagnostic purposes and were anonymized to protect the privacy of study participants. The final selection consisted of 25 sera from each of 5 consecutive winter seasons (2014–2019, 125 sera in total). The study protocol was approved by the medical ethical committee of Erasmus MC (MEC-2021-0027).

### Western blot

Western blot was used to assess the size of the G protein in virus stocks [39]. 400 µl virus stock was lysed with 100 µl 5X RIPA lysis buffer (125 mM Tris-HCl pH 7.6, 750 mM NaCl, 5% NP-40, 5% sodium deoxycholate, 0.5% SDS), phosphatase (Merck) and protease inhibitors (Merck) were added, centrifuged at 8000xg for 15 min, and the supernatant was stored at -80ºC. Proteins were separated using an Any-kD pre-cast SDS-polyacrylamide gel (BioRad) with a molecular marker to determine protein size (Precision Plus Protein Kaleidoscope Prestained Protein Standards, Bio-Rad). Proteins were transferred onto 20 μm PVDF membranes (Merck) by electroblotting for 1 h in transfer buffer (25 mM Tris, 192 mM glycine, and 20% methanol). The membranes were blocked in PBS with 10% [w/v] nonfat dried milk and 0.1% Tween-20 for 1 h at RT, incubated with a primary antibody against HRSV G (Abcam, clone RSV133, cat 94,966) and beta-actin (Santa Cruz Biotechnology, clone C4, cat SC-4778) in PBS 1% [w/v] nonfat dried milk and 0.1% Tween-20 for 2 h at room temperature and overnight at 4 ºC. Blots were washed 3 times at room temperature with PBS containing 0.1% Tween-20, incubated with rabbit anti-mouse Ig/HRP (DAKO, cat P026002-2) in PBS with 1% nonfat milk [w/v] and 0.1% Tween-20, washed again 3 times in PBS with 0.1% Tween-20, before storing in PBS at 4 ºC. Blots were developed with ECL Plus Western Blot Detection system kit (GE Healthcare, cat RPN2232) according to the manufacturer’s instructions and visualized using a Biorad Chemidoc.

### Focus reduction neutralization test in Vero cells

The F-specific moAb palivizumab (AstraZeneca, EU/1/99/117/003) and nirsevimab (AstraZeneca, EU/1/22/1689/004), and the G-specific moAb RSV133 (Abcam, Ab94966), and 131-2G (Merck Life Science, MAB858-2) were incubated at 10, 1, 0.1 and 0.01 µg/ml with ~ 500 TCID_50_ rHRSV-A11 or -B05 for 1 h at 37˚C. The mixtures were added to 4- to 5-day old monolayers of Vero cells and incubated for 2 days. Alternatively, a 2-fold dilution series starting at 1:32 of human or ferret serum was incubated in triplicate with ~ 1,250 TCID_50_ rHRSV-A11 or ~ 135 TCID_50_ rHRSV-B05. The WHO 1st international HRSV standard (NIBSC) was included in this assay. Virus-serum mixture was added to 4- to 5-day old monolayers of Vero cells and incubated for 2–3 days. Plates were scanned on a Typhoon laser-scanning platform and fluorescent foci were counted using ImageQuant. The counts were used to determine the 50% focus reduction neutralization titer (FRNT_50_) as described previously [40].

### Virus neutralization assay on AO at ALI

The moAb palivizumab, nirsevimab, RSV133, and 131-2G were incubated at 10, 1, 0.1 and 0.01 µg/ml with ~ 1,000 TCID_50_ rHRSV-A11 or -B05 for 1 h at 37˚C. The virus-antibody mixtures were added to 4–6 week differentiated AO at ALI. After 1 h, the virus-antibody mixtures were removed to restore the air-liquid interface. After 48 h, plates were scanned on a Typhoon laser-scanning platform as described before.

### Virus neutralization assay on AO at ALI^DAPT^

Palivizumab was incubated at 100, 10, 1, 0.1 µg/ml with ~ 600 TCID_50_ rHRSV-A11 or ~ 200 TCID_50_ rHRSV-B05 for 1 h at 37˚C. The virus-antibody mixtures were added to AO at ALI^DAPT^. After 1 h, the virus-serum mixtures were removed and plates were incubated for 72 h before scanning on a Typhoon laser-scanning platform. A selection of infant sera and palivizumab was tested for the presence of neutralizing antibodies using AO at ALI^DAPT^ cells obtained in suspension. The AO at ALI^DAPT^ were detached by adding 0.05 mM EDTA to the basolateral and apical compartment for 5–10 min. When the cells detached, single cell suspensions were seeded in U-bottom 96-well plates at 1,000 cells per well in 50 µl advanced DMEM supplemented with 10% fetal bovine serum, 100 IU penicillin + 100 µg streptomycin (Westburg) and 1x glutamax (Gibco). Duplicates of human sera were incubated with ~ 600 TCID_50_ rHRSV-A11 or ~ 200 TCID_50_ rHRSV-B05 in a 4-fold dilution series starting at a concentration of 1:32 (100 ug/ml for palivizumab) for 1 h at 37 ºC. Virus-serum mixture was added to AO at ALI^DAPT^ in suspension and after 3 days cells were screened for fluorescence.

### Virus neutralization assay on Ap-O AO

The moAb palivizumab, nirsevimab, RSV133, or 131-2G were incubated at 10, 1, 0.1, and 0.001 µg/ml with ~ 100 TCID_50_ rHRSV-A11 or -B05 for 1 h, after which the mixture was added to Ap-O AO. For human or ferret serum, a 3-fold dilution series starting at 1:8 was incubated in duplicate with ~ 100 TCID_50_ rHRSV-A11 or ~ 100 TCID_50_ rHRSV-B05. After 2 days, Ap-O AO were fixed for 20 min in 2% PFA, followed by staining with Hoechst (1:1,000) for 10 min. Ap- O AO were washed in PBS to remove Hoechst and PFA and transferred in 50 µl to black flat-bottom plates. 50 µl of 0.5% of warm low melting point agarose diluted in PBS was added to the wells, resulting in a 0.25% agarose solution to fix the Ap-O AO in place during scanning. We evaluated different methods for imaging and quantifying HRSV-infected Ap-O AO by using confocal microscopy, Typhoon imaging, Opera imaging, and CTL Immunospot imaging (Supplemental Fig. 1D-G). Quantification of fluorescent foci was optimal using the CTL Immunospot. The 50% neutralization titer (VNA_50_) was calculated using Graphpad Prism 10.1.1.

### Multiplex immunoassay

A serological bead-based multiplex immunoassay (MIA) to detect antibodies binding to HRSV-N, pre-fusion HRSV-F, post-fusion HRSV-F, HRSV-Ga and HRSV-Gb was performed as described previously [41].

### Indirect immunofluorescence staining

Transwell inserts were stained as previously described [37, 42]. Briefly, inserts were fixed in 4% (wt/vol) PFA for 30 min. Filters were washed twice, permeabilized with 0.2% Triton-X, and blocked in 10% NGS in DPBS for 30 min. Cells were incubated with moAb for 60 min in staining buffer containing 10% NGS and 2% (wt/vol) bovine serum albumin (BSA). Tight junctions were stained using anti-zona-occludens 1 (clone 1A12; Alexa Fluor 550; Santa Cruz Biotechnologies, 1:200), cilia were stained using anti-acetylated tubulin (clone 6-11B-1; Alexa Fluor 647; Santa Cruz Biotechnologies, 1:200) and the HRSV entry receptor was stained using a CX3CR1 antibody (clone 2A9-1; Alexa Fluor 488, Life Technologies/Invitrogen, 1:100). Hoechst was added during the last 10 min of the staining (Life Technologies/Invitrogen, catalog no. 10,150,888). Cells were washed three times with staining buffer and mounted in Prolong antifade mounting medium (Life Technologies/Invitrogen, catalog no. 9P36961). The cells were imaged on an LSM700 confocal microscope using ZEN software (Zeiss) and analyzed in Fiji [42]. Vero cells and Ap-O AO were stained in a similar matter.

### Flow cytometry

AO cultured at ALI in the presence or absence of DAPT were harvested by adding either Trypsin-EDTA (0.05%) phenol red (Thermofisher Scientific, catalog no 25,300,054), TryplE Express Enzyme (1x) no phenol red (Themofisher scientific, cat no 12,604,013), or 0.05 mM EDTA (0.05% in PBS, filter-sterilized) to the apical and/or basolateral compartment. Harvested cells were washed (400xg, 5 min) in FACS buffer (PBS with 0.05% BSA and 2 mM EDTA), stained with a CX3CR1 antibody (clone 2A9-1; Alexa Fluor 488, Life Technologies/Invitrogen, 1:100) for 30 min at room temperature, washed once (400xg, 5 min), and resuspended in FACS buffer. Samples were measured on a FACS Lyric (BD) and analyzed using FlowJo software.

### Statistics

The individual replicates or means are depicted in graphs. Experiments were performed in technical duplicates in one or more experiments. All analyses were performed using GraphPad Prism 10.1.1.

## Results

### G-specific neutralizing antibodies can exclusively be detected in cells expressing CX3CR1

**Fig. 1:**
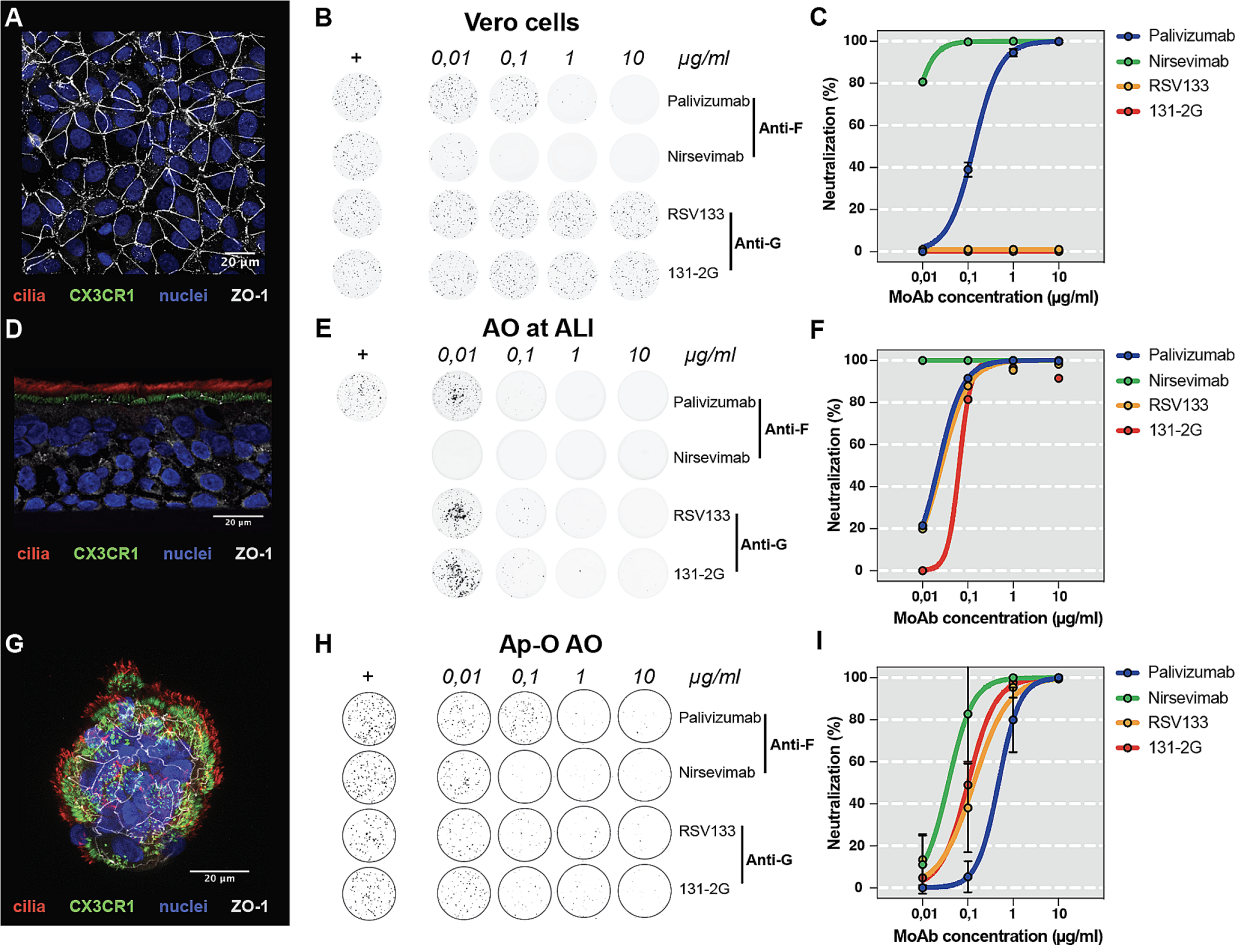
CX3CR1 expression and neutralization by HRSV F- and G-specific moAb on Vero cells, AO at ALI and Ap-O AO. (**A**) Vero cells were stained for the presence of cilia (red), CX3CR1 (green), nuclei (blue) and ZO-1/tight junctions (white). Images were made using a confocal laser scanning microscope with a Z-stack top view. (**B**) HRSV-specific moAb directed against the F (palivizumab and nirsevimab) or G protein (RSV133 and 131-2G) were tested in 4 different concentrations ranging from 10 µg/ml to 0.01 µg/ml against rHRSV-A11 and rHRSV-B05 (only A11 shown in the figure; HRSV stocks grown on HEp2 cells). Typhoon images were made after 48 h and representative wells are shown. (**C**) Foci of infection were counted using ImageQuant; percentage neutralization relative to the controls was plotted. Similar staining and VNA were performed for AO at ALI (**D-F**) and Ap-O AO (**G-H**). For imaging of cilia, CX3CR1, nuclei and ZO-1 in the AO at ALI a side view was taken. For Ap-O AO, images of the wells were made with the Immunospot and infected Ap-O AO were counted using the Immunospot software

To confirm that HRSV G-specific antibodies can only be detected in cells expressing the HRSV entry receptor CX3CR1, we stained Vero cells, AO at ALI, and Ap-O AO for the presence of CX3CR1 and measured the neutralizing capacity of F- and G-specific moAb on these cells. As previously described, CX3CR1 was absent in Vero cells (Fig. 1A), and only the HRSV F-specific moAb palivizumab and nirsevimab neutralized rHRSV-A11 and -B05 (Fig. 1B-C). In contrast to Vero cells, AO at ALI expressed CX3CR1 on the apical surface of respiratory epithelial cells at the base of the cilia (Fig. 1D). Both HRSV G- and -F specific moAb neutralized rHRSV-A11 and -B05 in these cultures (Fig. 1E-F).

To develop a high-throughput assay with primary airway cells in suspension, we initially tried to optimize AO at ALI cultures by adding the notch inhibitor DAPT, described to accelerate differentiation into ciliated cells [43]. Culturing AO at ALI in the presence of DAPT resulted in a thin cell layer consisting mostly of ciliated epithelial cells, compared to the pseudostratified layer formed by AO at ALI cultured without DAPT (Supplemental Fig. 2A). To obtain single cell suspensions of CX3CR1^+^ ciliated epithelial cells for VNA, we evaluated several methods to detach cells without affecting expression of the surface receptor, using either (1) trypsin, (2) tryplE, or (3) 0.05 mM EDTA. EDTA detachment resulted in optimal detachment while retaining cilia and expression of CX3CR1 (Supplemental Fig. 2B-C). Surprisingly however, HRSV could not be neutralized when using these single cell suspensions in VNA, even though palivizumab treatment did result in neutralization on AO at ALI cultured with DAPT (Supplemental Fig. 2D).

Next, we investigated Ap-O AO as a model for an AO-based high-throughput VNA. After expansion and differentiation, Ap-O AO maintained their 3D structure with cilia on the outside, leading to migration throughout the well (Fig. 1G, Supplemental video 1). CX3CR1 was detected at the base of the cilia. As expected, Ap-O AO were susceptible to HRSV infection (Supplemental Fig. 1D). Both F- and G-specific moAb neutralized HRSV-A11 and -B05 in Ap-O AO, but at higher concentrations compared to Vero cells (Fig. 1H-I).

### HRSV stocks grown on immortalized or primary cells are equally susceptible to neutralization

**Fig. 2:**
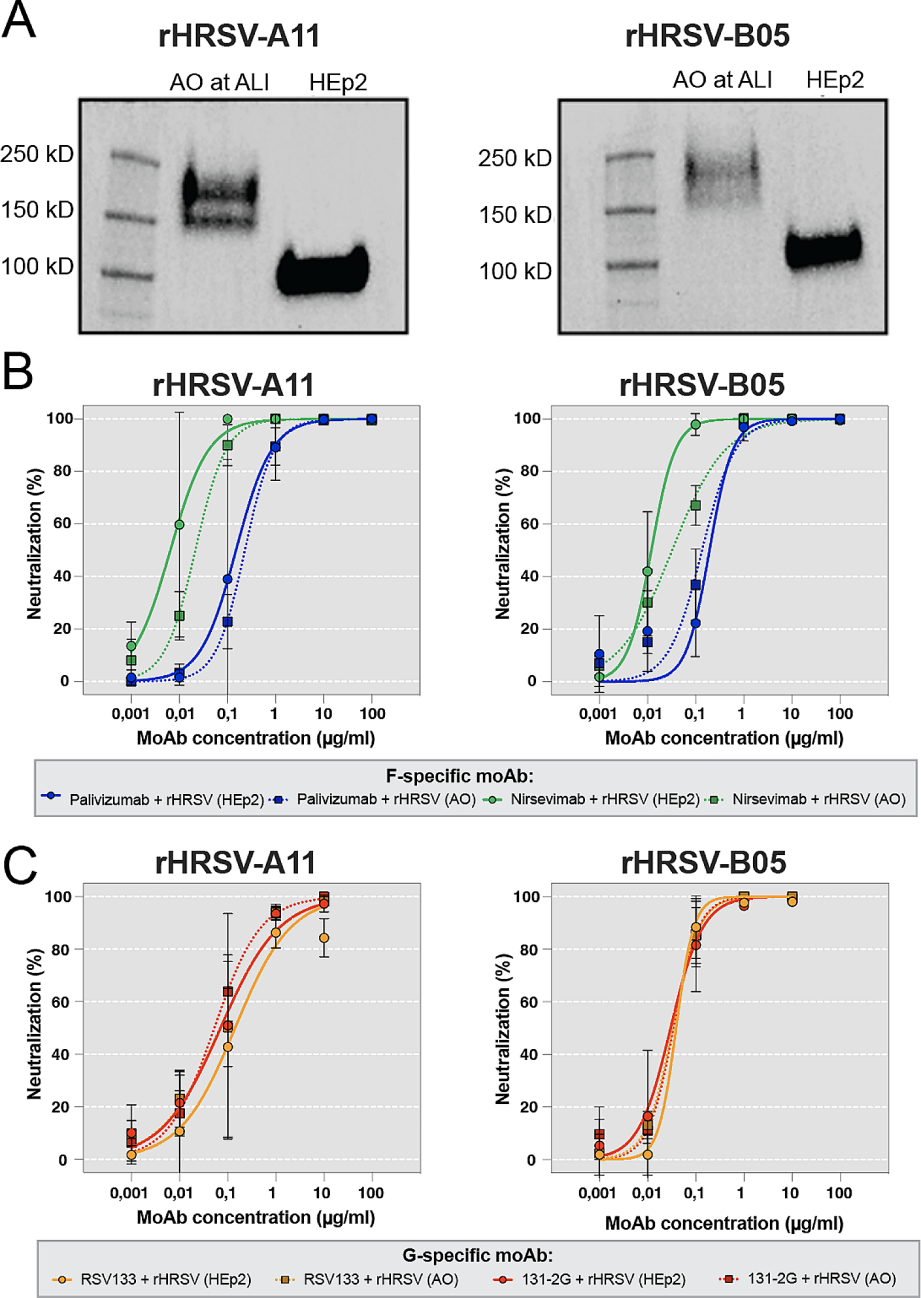
Comparison of rHRSV-A11 and -B05 stocks grown on HEp2 cells or AO at ALI. rHRSV-A11 and -B05 stocks were produced on HEp2 or AO at ALI. (**A**) The size of the G protein was analyzed by gel electrophoresis and immunoblotting with a G-specific mouse monoclonal. (**B**) Susceptibility of rHRSV-A11 and -B05 stocks grown on HEp2 or AO at ALI to HRSV F-specific moAb on Ap-O AO. (**C**) Susceptibility of rHRSV-A11 and -B05 stocks grown on HEp2 or AO at ALI to HRSV G-specific moAb on Ap-O AO. VNA on Ap-O AO was imaged and counted using the Immunospot

Based on previously reported discrepancies in glycosylation of the G protein between HRSV grown on immortalized cells versus differentiated airway epithelial cultures, we cultured rHRSV-A11 and -B05 virus stocks on either HEp2 cells or AO at ALI, and compared the susceptibility of stocks obtained on different source cells to neutralization. HRSV stocks grown on AO at ALI indeed had a larger G protein (~ 150–200 kDa) compared to HRSV stocks grown on HEp2 cells (~ 100 kDa) (Fig. 2A) [30]. To determine differences in susceptibility to neutralization, we incubated rHRSV-A11 and -B05 grown on HEp2 or AO at ALI with different concentrations of F- and G-specific moAb, and inoculated Ap-O AO with the moAb-virus mixtures. Similar to the data shown in Fig. 1, nirsevimab neutralized HRSV 10-fold better compared to palivizumab. However, both the HEp2 and AO at ALI grown stocks were similarly susceptible to neutralization by F-specific moAb (Fig. 2B). The G-specific moAb RSV133 and 131-2G were equally potent against rHRSV-A11 and -B05, and no differences were observed between neutralization of the HEp-2 and AO at ALI stocks (Fig. 2C**)**. In conclusion, the larger G protein, presumably caused by different glycosylation patterns, did not alter the susceptibility of HRSV to neutralization by these specific moAb. In subsequent experiments, we used cell line-grown HRSV stocks in cell line-based VNA, and AO at ALI-grown HRSV stocks on AO-based VNA and titrated on the same cells as employed in the assay.

### HRSV subgroup A- or B neutralizing antibodies are detectable on Vero cells and Ap-O AO

**Fig. 3:**
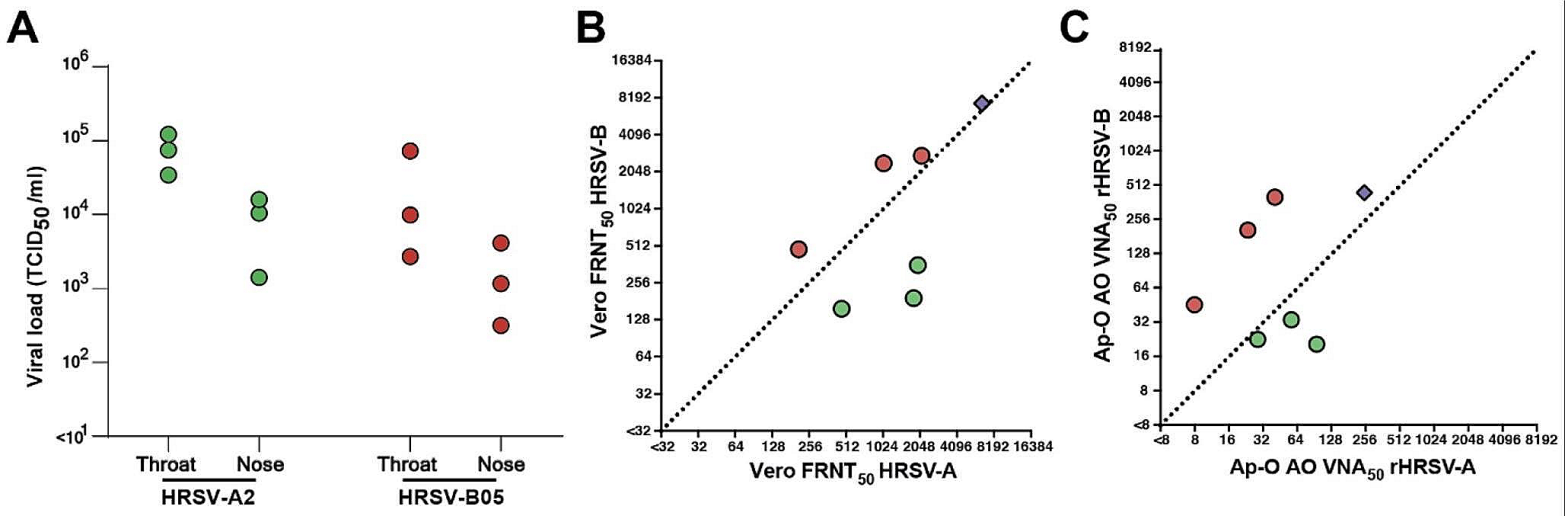
Neutralizing antibodies induced by a single HRSV subgroup A or B infection in ferrets measured via FRNT on Vero cells and VNA on Ap-O AO. Ferrets were inoculated intranasally with 10^5^ TCID_50_ HRSV-A2 (*N* = 3) or HRSV-B05 (*N* = 3). (**A**) At 3 DPI viral loads were determined by endpoint titration of nose and throat swabs. (**B**) FRNT on Vero cells was performed with ferret sera obtained 21 DPI to determine the FRNT_50_ against rHRSV-A11 (x-axis) and -B05 (y-axis). (**C**) VNA on Ap-O AO was performed with ferret sera obtained 21 DPI to determine the VNA_50_ against rHRSV-A11 (x-axis) and -B05 (y-axis). The diagonal line is a visual aid at identical titers. The WHO 1st International Standard antiserum to HRSV was included in the Vero FRNT (**B**) and Ap-O AO (**C**) and is indicated by a purple diamond

To obtain sera resulting from single HRSV-A or -B infection for assay validation purposes, we inoculated ferrets with either HRSV-A2 or -B05 and euthanized these animals at 21 days post-inoculation (DPI). Productive HRSV infection was confirmed by virus isolation from the nose and throat at 3 DPI (Fig. 3A). Neutralizing antibodies were measured by both FRNT on Vero cells (Fig. 3B) and VNA on Ap-O AO (Fig. 3C). In both FRNT on Vero and VNA on Ap-O AO, neutralizing antibodies were measurable, and titers were higher against the same subtype virus compared to the heterologous virus. A human standard antiserum to HRSV was included as a positive control and neutralized both viruses at similar titers (purple diamond, Fig. 3B-C). The VNA on Ap-O AO resulted in lower neutralizing antibody levels compared to the FRNT on Vero cells (geometric mean titer (GMT) 16,4-fold lower).

### HRSV-specific antibodies in human serum detected with VNA based on Ap-O

Next, we made a selection of 125 serum samples obtained from infants below the age of 3 years collected during 5 consecutive winter seasons (2014–2019). The median age of the infants was similar over the 5 seasons (Supplemental Fig. 3). We selected sera from infants below the age of three as we considered it likely that these had experienced a single HRSV infection (either subgroup A or B), and a discrimination between HRSV subgroup A or B neutralization could be measurable. As reported by the Dutch national institute for public health and the environment (RIVM), HRSV subgroup B was most prevalent in seasons 2015–2016, 2017–2018, and 2018–2019, whereas HRSV subgroup A was more prevalent in the winter season of 2016–2017 [44–47]. No data was available for season 2014–2015.

Infant sera were screened for the presence of binding antibodies to the pre-F, post-F, nucleoprotein (N), Ga and Gb protein by multiplex immunoassay (Supplemental Fig. 4A-C). Pre-F antibodies correlated well with post-F antibodies (r^2^ = 0.93, Supplemental Fig. 4A). There was a reduced correlation between antibodies targeting Ga and those targeting Gb (r^2^ = 0.44, Supplemental Fig. 4B). Some sera seemed to preferentially bind either Ga or Gb by showing higher levels against one of the variants. Nucleoprotein (N)-specific antibodies correlated with both pre-F (r^2^ = 0.87) and post-F (r^2^ = 0.87) binding antibodies (Supplemental Fig. 4C).

**Fig. 4:**
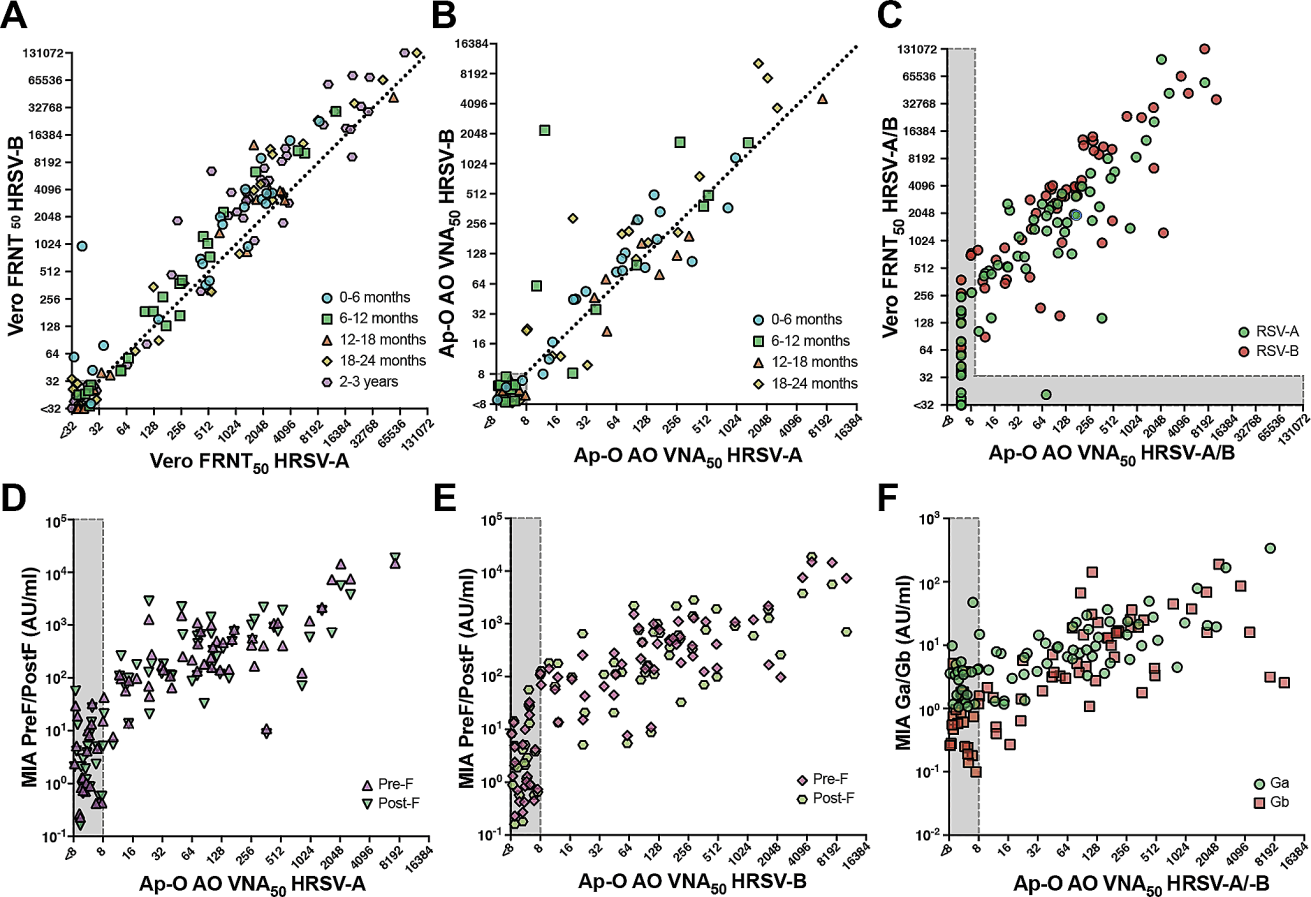
Comparison of HRSV-specific antibody levels in infant sera measured by FRNT on Vero cells and VNA on Ap-O AO. (**A**) rHRSV-A11 (x-axis) and -B05 (y-axis) neutralizing antibodies were measured in a selection of sera from infants (*n* = 125) (aged < 36 months) by FRNT on Vero cells. Sera are color-coded by age group. (**B**) Neutralizing antibody levels in identical sera as in panel A (excluding 2–3-year-old infants, *N* = 73) were tested by VNA on Ap-O AO and color-coded by age group. (**C**) Correlation between FRNT on Vero cells and VNA on Ap-O AO. In green VNA_50_ titers against HRSV-A11 compared to FRNT_50_ titers against HRSV-A11, in red VNA_50_ and FRNT_50_ titers against HRSV-B05. (**D, E**) Antibodies binding the pre- and post-fusion conformation of F were measured in MIA (y-axis) and correlated to VNA_50_ titers (x-axis) against (**D**) rHRSV-A11 and (**E**) rHRSV-B05. (**F**) Antibodies binding the G protein of HRSV-A (Ga) were correlated to the rHRSV-A11 VNA_50_ titers of the infant sera (green), and antibodies binding the G protein of HRSV-B (Gb) to the rHRSV-B05 VNA_50_ titers (red). The grey boxes indicate measurements below the limit of detection

Next, we determined the neutralizing capacity of the infant sera in a side-by-side comparison between the FRNT on Vero cells and VNA on Ap-O AO, using both rHRSV-A11 and -B05 (Fig. 4A-B). A total of 23/125 sera did not neutralize HRSV (18,4%), whereas 102/125 sera were HRSV-seropositive by FRNT on Vero cells (81,6%, Fig. 4A). We observed that sera from infants aged 2–3 years had high titers (Fig. 4A, pink hexagon points on plot) and assumed that these infants experienced multiple HRSV infections. These sera were excluded from further analysis. Interestingly, neutralizing antibody levels were also high in infants 0–6 months of age, potentially reflecting the presence of maternal antibodies (Supplemental Fig. 5A-B).

A total of 73 sera was tested by VNA on Ap-O AO, which resulted in 22/73 HRSV-seronegative sera (30.1%) and 51/73 HRSV-seropositive sera for neutralization of at least one HRSV subtype (69.9%, Fig. 4B). For both neutralization of HRSV-A11 and -B05, the FRNT and VNA correlated well (rHRSV-A11 r^2^ = 0.75, rHRSV-B05 r^2^ = 0.78, Fig. 4C), but neutralizing antibody levels measured on Ap-O AO were lower than titers obtained on Vero cells (GMT 14.0-fold lower). FRNT titers for both rHRSV-A11 and rHRSV-B05 correlated with the pre-F (rHRSV-A11 r^2^ = 0.86, rHRSV-B05 r^2^ = 0.86) and post-F (rHRSV-A11 r^2^ = 0.79, rHRSV-B05 r^2^ = 0.79) binding antibody levels measured by MIA (Supplemental Fig. 4D-E). This is similar for VNA titers with pre-F (rHRSV-A11 r^2^ = 0.65, rHRSV-B05 r^2^ = 0.65) and post-F (rHRSV-A11 r^2^ = 0.61, rHRSV-B05 r^2^ = 0.60) binding antibody levels, although the MIA appeared more sensitive than VNA (Fig. 4D-E). In addition, Ga-specific binding antibody levels correlated with VNA rHRSV-A11 titers (r^2^ = 0.54), and Gb-specific binding antibody levels correlated with VNA rHRSV-B05 titers (r^2^ = 0.53, Fig. 4F). Similar correlations were found when comparing MIA Ga- and Gb-specific binding antibody levels to rHRSV-A11 and rHRSV-B05 FRNT_50_, respectively (rHRSV-A11 r^2^ = 0.39, rHRSV-B05 r^2^ = 0.47, Supplemental Fig. 4F). In contrast to the sera obtained from ferrets that experienced a single HRSV infection, we could not distinguish between preferential HRSV-A- or -B-specific neutralization in sera obtained from seasons in which either subtype was more prevalent (Supplemental Fig. 5C-D). Our findings reveal successful quantification of HRSV neutralizing antibodies on Ap-O AO, correlating with traditional assays such as FRNT and MIA.

## Discussion

Here, we showed that Ap-O AO express CX3CR1, are susceptible to HRSV infection, and are suitable targets to measure both HRSV F- and G-specific neutralizing antibodies. We optimized the quantification of HRSV-infected Ap-O AO using an immune monitoring platform (CTL Immunospot) and showed in a side-by-side comparison with a FRNT on Vero cells that neutralizing antibody levels observed in the VNA on Ap-O AO correlated well. The Ap-O AO culture model is promising for neutralization assays, because it is scalable, affordable, and can be used in high throughput.

The ability to accurately measure functional antibodies to HRSV is crucial to better understand the immune response after infection or (passive) immunization. Currently, there is no harmonized approach to measure HRSV-specific neutralizing antibodies [13], but it is thought that neutralization assays like a FRNT are robust and reliable, especially when using viruses that express a fluorescent reporter protein [17, 19]. Here, we further optimized the FRNT by using clinical-based RSV strains for both subtype A and B (instead of the often-used lab-adapted viruses) and obtained consistent results that correlated well with a validated bead-based MIA assay. Using moAb, we showed that immortalized cell line-based neutralization assays detect HRSV F-specific neutralization, but fail to detect neutralization mediated by G-specific antibodies, as was described before [48, 49]. HRSV G-specific antibodies have been measured in neutralization assays performed on well-differentiated HAE at ALI [23, 28, 29, 48]. Here, we showed that the presence of CX3CR1 in differentiated respiratory epithelial cells allows for the detection of both HRSV F- and G-specific antibodies, although it cannot be excluded that other proteins play a role as well. Introducing CX3CR1 expression into a cell line is a potential strategy; however, immortalized cell lines have other ‘receptors’ than the natural target cells, which may inaccurately reflect the in vivo neutralizing capacity of HRSV-specific antibodies.

All approved prophylaxis against HRSV rely on the F protein (mostly the pre-fusion conformation) as an antigen, but our current understanding of the contribution of G-specific antibodies to clinical protection from HRSV disease is limited. To measure G-specific antibodies, we decided to develop a neutralization assay on primary cells. We have previously shown that primary respiratory epithelial cells, both from the upper and lower respiratory tract, and either commercially obtained or differentiated from AOs, sustain infection with subgroup A and B HRSV. We concluded that this is a valuable model to study HRSV-specific immune responses, which should be developed further [37]. Here, in our initial approach to create a high-throughput AO-based VNA that could also measure G-mediated neutralization, we attempted to re-direct differentiation of AO at ALI towards ciliated epithelial cells using DAPT, detach these cells, and perform a VNA in suspension. Although AO at ALI^DAPT^ suspension cultures were susceptible to HRSV infection, we could not neutralize HRSV even after pre-incubation with high concentrations of palivizumab or polyclonal ferret or human antisera. We hypothesize that was due to alternative virus entry routes that are independent of receptor interaction, possibly at the basolateral side of the cells in suspension or infection of the basal cells that are now also accessible and of which infection by HRSV has been described before [50]. This could potentially also involve macropinocytosis, a mechanism in which cells internalize HRSV containing droplets, an entry mechanism that was previously described for HRSV [51].

Our focus shifted towards Ap-O AO, which distinguishes itself by faster differentiation compared to AO when cultured at ALI. This method offers scalability and cost-effectiveness making it suitable for high-throughput applications. Ap-O AO can be cultured in suspension, but unlike the cultures described above, viral access remains restricted to the apical surface of the epithelial cells [32]. Ap-O AO proved susceptible to HRSV infection, but the accurate detection of HRSV infection by EGFP expression in 3D Ap-O AO was challenging. Imaging via Typhoon, our preferred method for FRNT on Vero cells, did not allow quantification, which is crucial to determine VNA_50_ titers. As an alternative, we considered a confocal-based system (Opera), capable of accurately detecting the number of infected cells per Ap-O AO, but requiring long processing times. We found that an immune monitoring platform (CTL Immunospot) facilitated rapid imaging while maintaining high-quality and accurate output. Our optimized Ap-O AO VNA demonstrated suitable for detecting both F- and G-specific mediated neutralization at a large scale.

Because the main antigenic differences between the HRSV-A and -B subgroups are in the G protein, we expected that the detection of HRSV G-specific antibodies by VNA on Ap-O AO could reveal if an individual was previously infected with HRSV subtype A or B [3, 52, 53]. Our data showed that HRSV-A or B-specific neutralization could be distinguished using sera from ferrets with a single HRSV infection, using either the FRNT on Vero or VNA on Ap-O AO. This indicates that antigenic differences between different HRSV strains can even be detected in the F protein, in contrast to the frequently asserted minimal genetic variations within the F proteins across all HRSV strains. Since mono-specific sera from ferrets allowed antigenic discrimination, antigenic cartography, as previously performed for influenza virus and SARS-CoV-2 [54–57], could be good approach to visualize the antigenic evolution of HRSV.

Interestingly, the detection of HRSV G-specific mediated neutralization on Ap-O AO in sera from infants expected to have been infected with HRSV once, did not lead to preferential neutralization of either HRSV-A or -B. In addition, detecting specific HRSV-A or -B subtypes in 0–6 month-old infants may be complicated by the presence of maternal antibodies, given that mothers usually have experienced multiple HRSV infections. The use of virus stocks grown on primary cells did not seem to change this result as we did not detect significant differences in moAb neutralization when comparing the AO-grown HRSV stocks to HEp-2-grown HRSV stocks. This observation suggests that infection with either subtype HRSV-A or -B elicits the production of neutralizing antibodies that target both sybtypes, indicating a cross-reactive immune response. We consistently measured lower neutralizing antibody levels on Ap-O AO compared to levels measured on Vero cells. This could be related to the previously reported difference in relative infectivity of HRSV in Vero cells and Ap-O AO, especially with the use of either cell line-grown or AO-grown HRSV stocks [30]; the fact that Vero cells lack an IFN response could be another contributing factor [58–60]. The use of different neutralization assays could contribute to a better understanding of the mechanisms of entry used by HRSV. We speculate that the lower neutralizing antibody titers measured on Ap-O AO could be more reflective of the genuine protective antibodies in vivo, keeping in mind that we are not protected from HRSV re-infections throughout our lives [61].

In conclusion, we developed a VNA for HRSV by using an AO-based cell model and AO-grown clinical-based viruses, allowing detection of both HRSV F- and G-specific neutralizing antibodies. This assay could serve multiple purposes: (1) elucidate the in vivo entry mechanisms of HRSV, (2) assess the potency of HRSV G-specific moAb, (3) understand the contribution of F- and G-specific antibodies to neutralization, and thereby (4) contribute to a better understanding of correlates of protection. While our present study focused on the neutralizing capacity of infant sera, antibody functionality is not restricted to neutralization. Especially for HRSV, Fc-mediated functionality was shown to be a potential correlate of protection in mice, non-human primates and humans [62–64]. The Ap-O AO system described here holds potential for assessing Fc-mediated functionalities. A comprehensive assessment of all functional aspects of antibodies, i.e. neutralizing and Fc-mediated responses, will be required to unveil the full functionality of HRSV-specific antibodies in the future. This is crucial in the evaluation of vaccine-mediated responses.

## Electronic supplementary material

Below is the link to the electronic supplementary material.

Supplementary Material 1

Supplementary Material 2

Supplementary Material 3

Supplementary Material 4

Supplementary Material 5

Supplementary Material 6

Supplementary Material 7

## Data Availability

The datasets generated during and/or analysed during the current study are not publicly available, but are available from the corresponding author on reasonable request.
