# Supplementary figures and images for: Virus neutralization assays for human respiratory syncytial virus using airway organoids
Source: Cell Mol Life Sci. 2024 Jun 17;81(1):267. doi: 10.1007/s00018-024-05307-y (PMC11335194; doi:10.1007/s00018-024-05307-y)

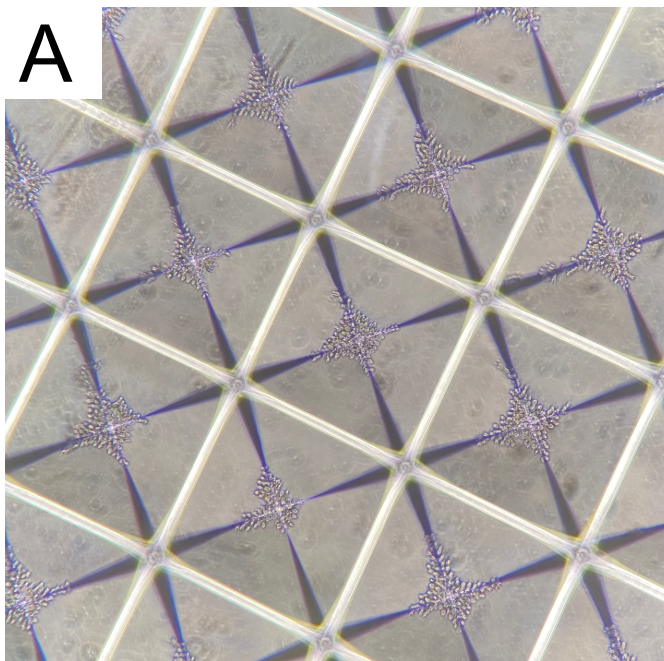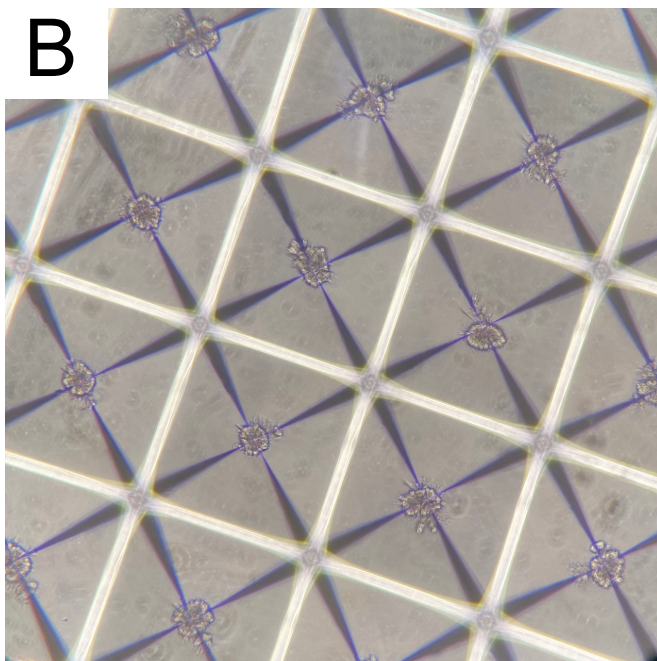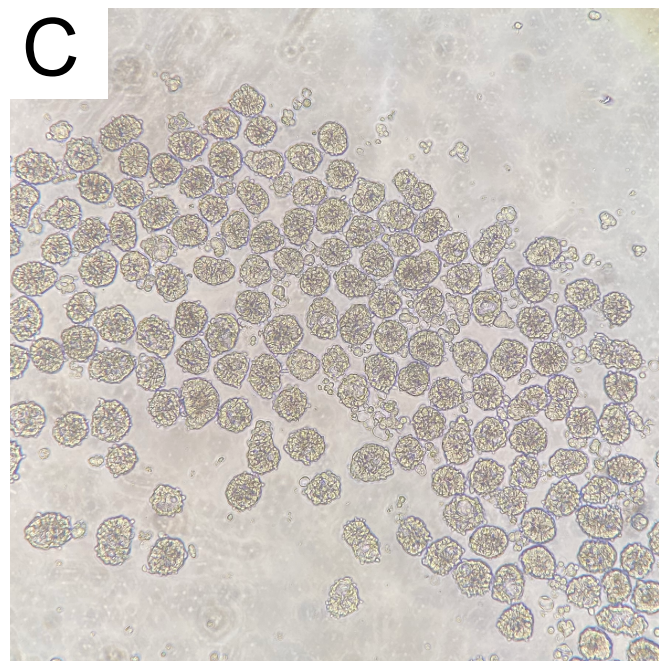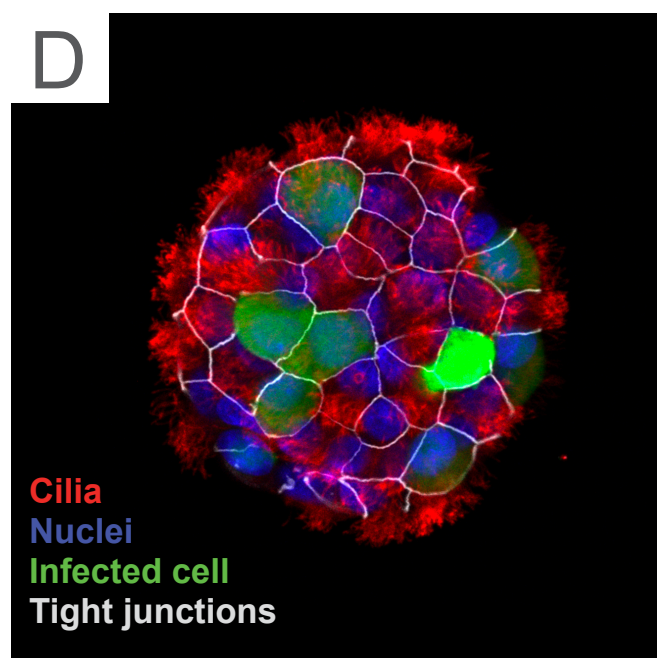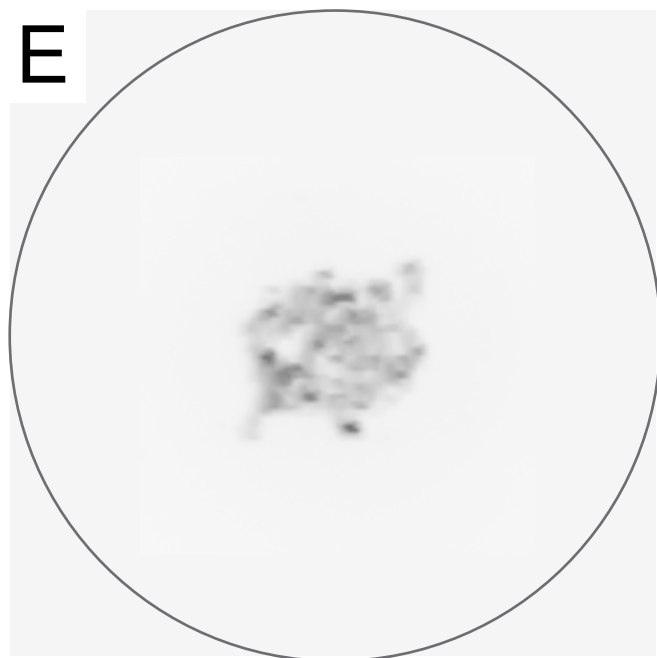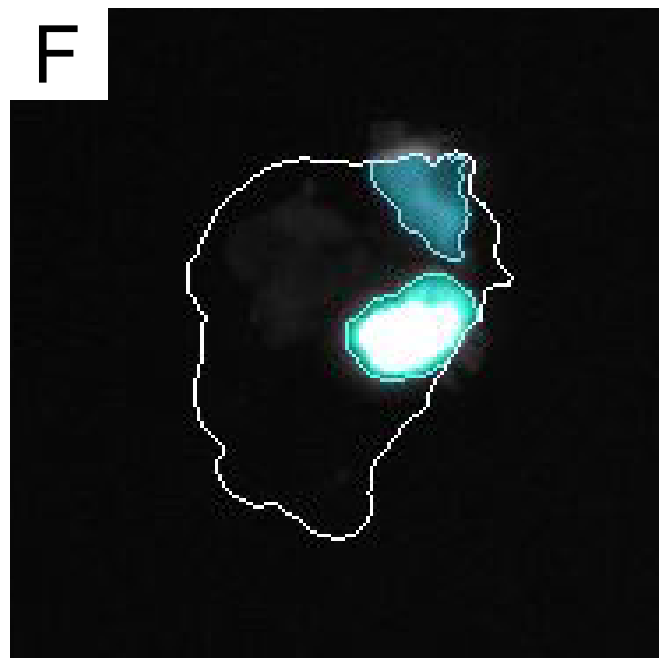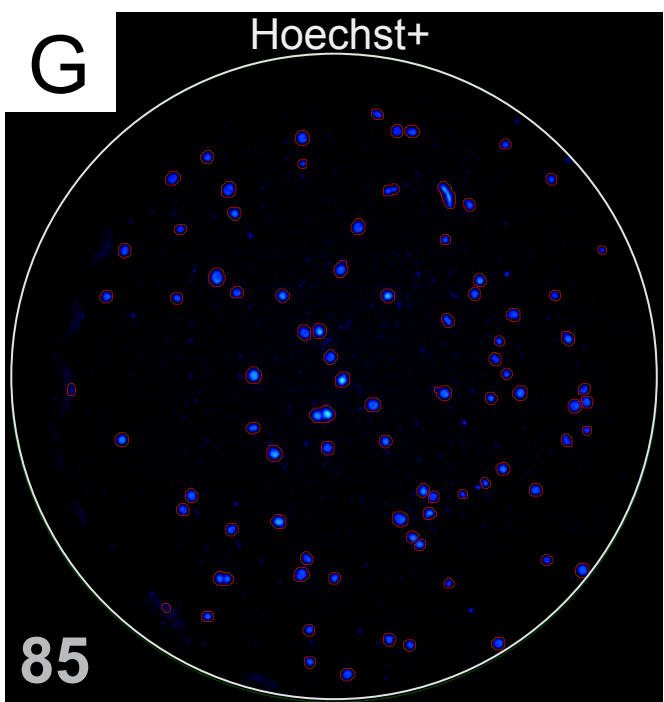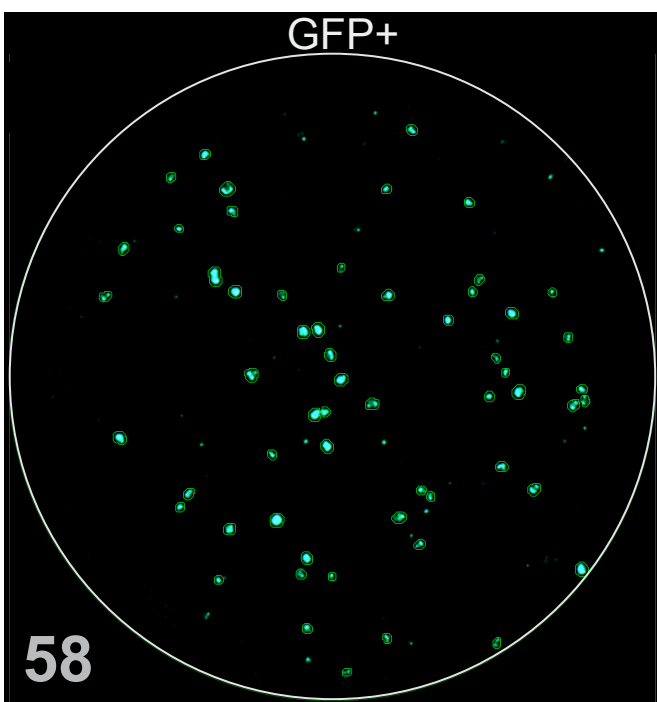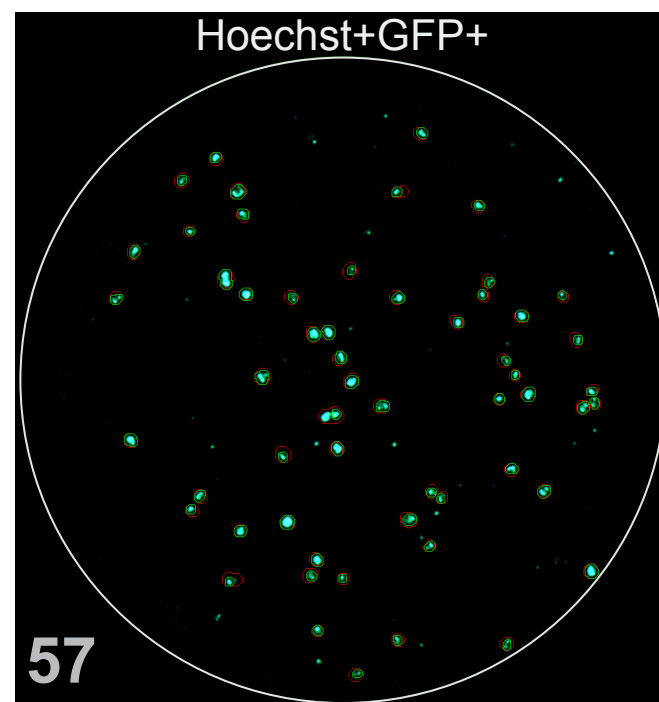

Supplement: Supplementary file 2 — Supplementary Material 2 [file 18_2024_5307_MOESM2_ESM.pdf]

**A**

No DAPT

DAPT

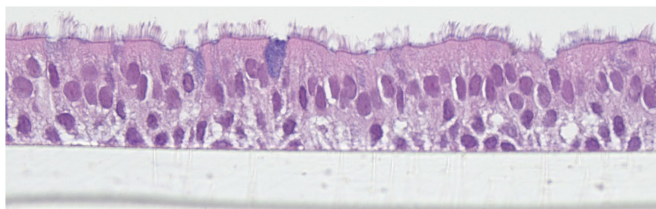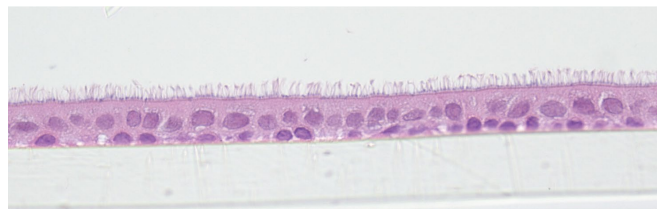**B**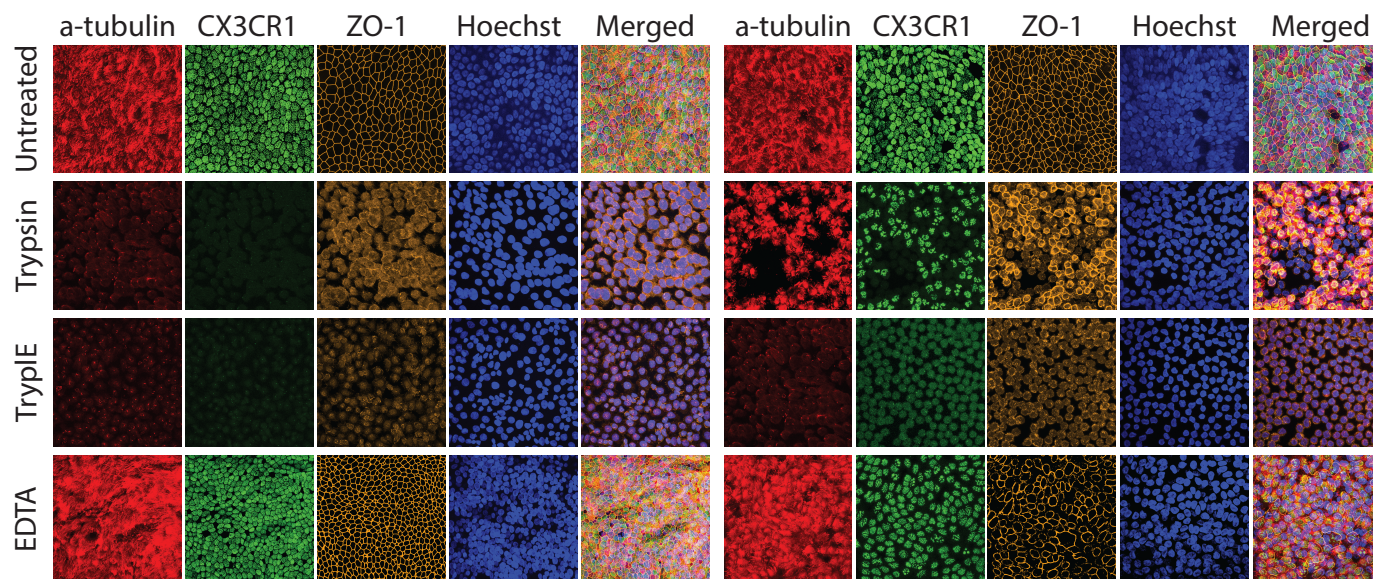**C**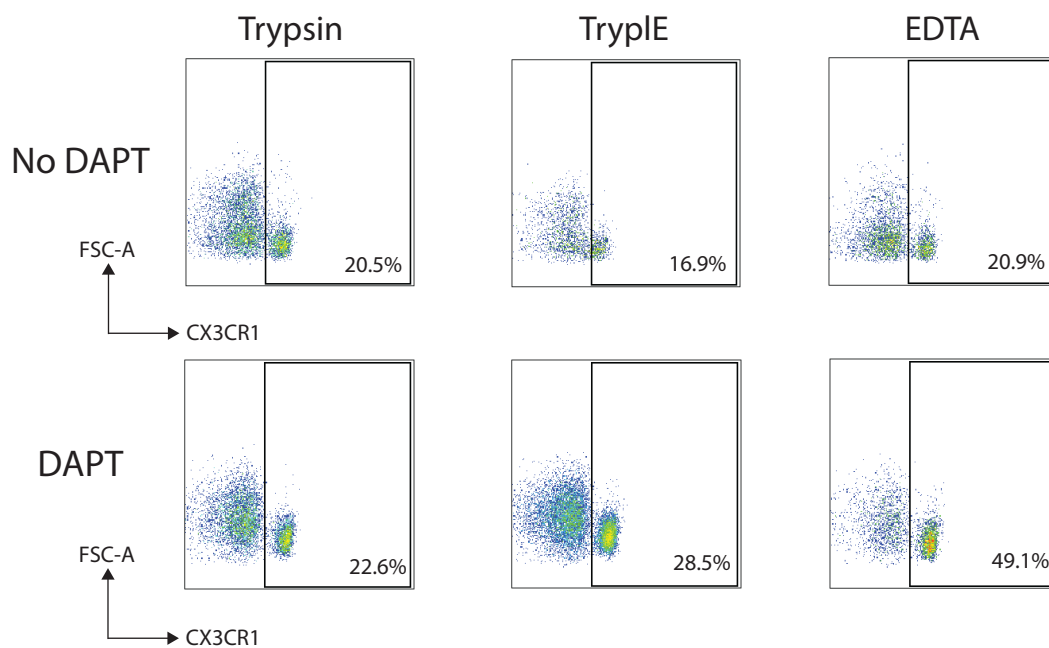**D**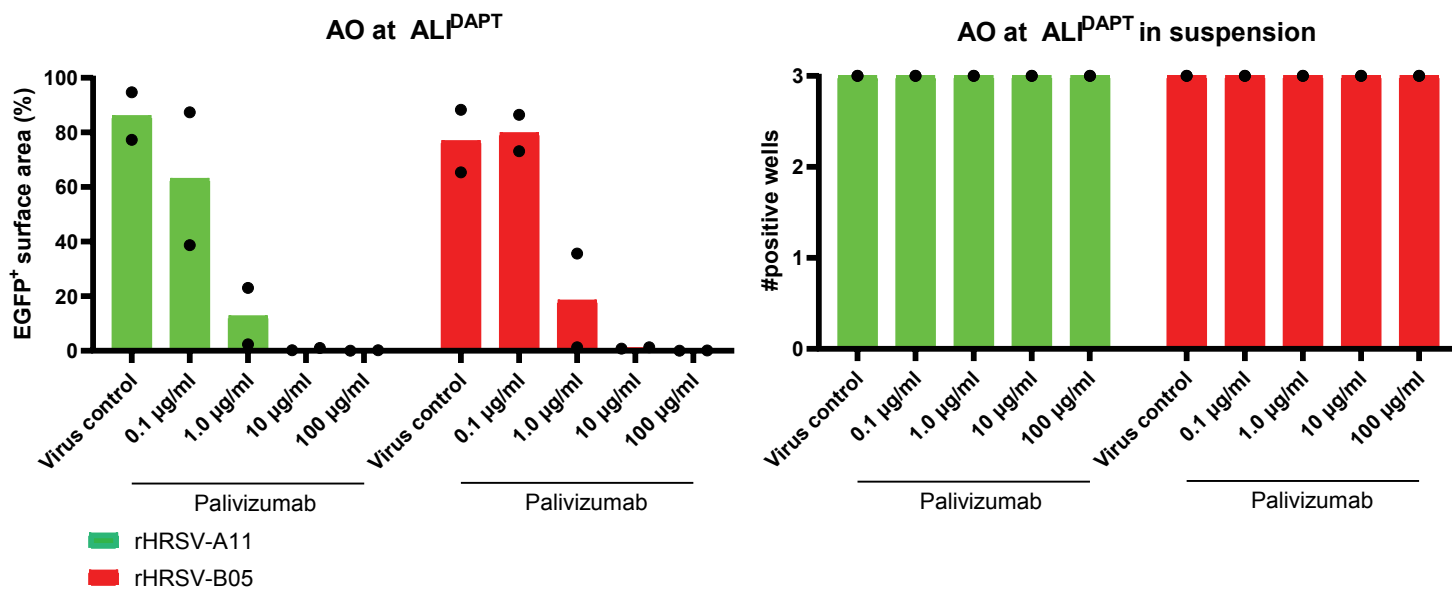

Supplement: Supplementary file 3 — Supplementary Material 3 [file 18_2024_5307_MOESM3_ESM.pdf]
