# Supplementary material for: Virus neutralization assays for human respiratory syncytial virus using airway organoids
Source: Cell Mol Life Sci. 2024 Jun 17;81(1):267. doi: 10.1007/s00018-024-05307-y (PMC11335194; doi:10.1007/s00018-024-05307-y)
Supplement: Supplementary file 7 — Supplementary Material 7 [file 18_2024_5307_MOESM7_ESM.docx]

SUPPLEMENTARY FIGURES

***Supplemental figure 1. Culture and optimization of imaging of Apical Out Airway Organoids (Ap-O AO).*** *(A) Single cell primary bronchial epithelial cells were generated from 3D bronchial airway organoids in Matrigel after incubation in tryplE and mechanical disruption. Single cells were seeded in an aggrewell plate. (B) After 2-4 days, the seeded cells formed small clumps and were transferred to a flat bottom 24-well plate. (C) During the following 1-2 weeks, the clumps differentiated and displayed cilia on the outside. (D) HRSV-infected differentiated Ap-O AO (HRSV-infected cells in green) were fluorescently stained for cilia (red), nuclei (blue), and tight junctions (white). Infected Ap-O AO were quantified using the Typhoon (E), Opera (F) or CTL Immunospot (G). The CTL immunospot counted the amount of Ap-O AO (stained blue, left), the amount of infected Ap-O AO (stained green, middle), and the overlay quantified infected Ap-O AO (right). CTL immunospot counts were used to calculate the VNA_50_.*

***Supplemental figure 2. Histology, indirect immunofluorescence and flow cytometry of AO at ALI cultured with or without DAPT and palivizumab neutralization on AO at ALI^DAPT^ and AO at ALI^DAPT^ in suspension.*** *(A) AO at ALI were grown with or without DAPT, fixed in formalin and embedded in paraffin for H&E staining. (B) AO at ALI cultures with or without DAPT were treated with trypsin, tryplE or EDTA (0.05 mM), fixed in 2% paraformaldehyde, and stained for CX3CR1 (green), zona-occludens 1 (ZO-1), acetylated α-tubulin (cilia, red) and Hoechst (nuclei, blue). (C) AO at ALI were grown with or without DAPT, detached with trypsin, tryplE or EDTA (0.05 mM), and stained for CX3CR1 and measured by flow cytometry. Representative images are shown, the percentage of CX3CR1 expressing cells is shown in the rectangular gate. (D) Palivizumab (100, 10, 1 and 0.1 μg/ml) neutralized AO-grown rHRSV-A11 or rHRSV-B05 when VNA were performed on AO at ALI^DAPT^ cultures, but not when single cell suspensions of these cultures were used. Experiments were performed either in duplicate or triplicate, and the mean and individual values are shown.*

***Supplemental figure 3. Age distribution of infant sera obtained between 2014-2019.*** *Sera from 125 infants were included in this study; N=25 sera in 5 consecutive seasons were collected. Median age per season is indicated by the dotted line. Symbols are color-coded based on age. Pie chart represents the number of serum samples per age group per season.*

***Supplemental figure 4. HRSV-specific antibodies in infant sera measured by multiplex immunoassay (MIA) and compared to neutralizing antibodies measured by FRNT on Vero cells.*** *Antibodies binding (A) the pre- and post-fusion conformation of F, or (B) the G protein of HRSV-A (Ga) or HRSB-B (Gb), were measured by MIA. Sera are color-coded by age group. (C) HRSV N-specific antibodies correlated to antibodies binding pre-F and post-F. Pre-F- and post-F-specific antibodies correlated to (D) rHRSV-A11 FRNT_50_ and (E) rHRSV-B05 by FRNT_50_. (F) Antibodies binding Ga correlated to the rHRSV-A11 FRNT_50_ titers (green), and antibodies binding Gb to the rHRSV-B05 FRNT_50_ titers (red). Grey boxes indicate measurements below the lower limit of detection.*

***Supplemental figure 5. HRSV-specific neutralizing antibodies measured in infant sera depicted per age group and per season.*** *(A) The geometric mean of FRNT_50_ titers on Vero cells depicted per age group for rHRSV-A11 (round symbols) and for rHRSV-B05 (triangle symbols). (B) The geometric mean of VNA_50_ titers on Ap-O AO depicted per age group for rHRSV-A11 (round symbols) and for rHRSV-B05 (triangle symbols). Sera from infants were tested by (C) FRNT on Vero cells and (D) VNA on Ap-O AO as shown in figure 4. Here, sera are color-coded based on the season in which they were collected. The grey boxes indicate measurements below the limit of detection.*
